# *catena*-Poly[[[dipyridine­copper(II)]-μ-2,3,5,6-tetra­methyl­benzene-1,4-di­carboxyl­ato] monohydrate]

**DOI:** 10.1107/S1600536810036913

**Published:** 2010-09-25

**Authors:** Xiaoqin Hu

**Affiliations:** a Department of Chemistry, North University of China, Taiyuan, Shanxi 030051, People’s Republic of China

## Abstract

In the title complex, {[Cu(C_12_H_12_O_4_)(C_5_H_5_N)_2_]·H_2_O}_*n*_, the Cu^II^ ion lies on an inversion center and is coordinated by two O atoms from two 2,3,5,6-tetra­methyl­benzene-1,4-dicarboxyl­ate (TBDC) ligands and two N atoms from two pyridine ligands in a slightly distorted square-planar environment. The TBDC ligands act as bridging ligands, forming chains along [110]. These chains are further linked into a two-dimensional network *via* inter­molecular O—H⋯O hydrogen bonds. The solvent water mol­ecule lies on a twofold rotation axis.

## Related literature

For related structures, see: Chun *et al.* (2005 ▶); Diniz *et al.* (2002 ▶).
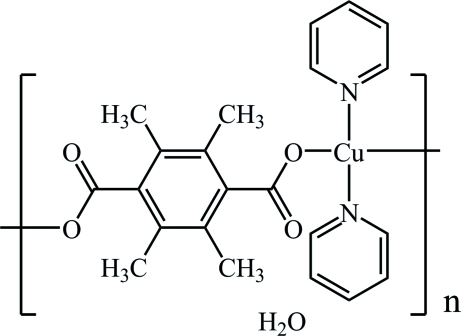


## Experimental

### 

#### Crystal data

[Cu(C_12_H_12_O_4_)(C_5_H_5_N)_2_]·H_2_O
*M* *_r_* = 459.98
Monoclinic, 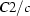

*a* = 13.3280 (8) Å
*b* = 17.1434 (11) Å
*c* = 10.7390 (7) Å
β = 108.481 (1)°
*V* = 2327.2 (3) Å^3^
*Z* = 4
Mo *K*α radiation
μ = 0.97 mm^−1^
*T* = 298 K
0.15 × 0.10 × 0.08 mm

#### Data collection

Bruker SMART CCD diffractometer
Absorption correction: multi-scan (*SADABS*; Sheldrick, 1996 ▶) *T*_min_ = 0.868, *T*_max_ = 0.926
6747 measured reflections
2594 independent reflections
2283 reflections with *I* > 2σ(*I*)
*R*_int_ = 0.017

#### Refinement

*R*[*F* ^2^ > 2σ(*F* ^2^)] = 0.029
*wR*(*F* ^2^) = 0.086
*S* = 1.06
2594 reflections
142 parameters
H atoms treated by a mixture of independent and constrained refinement
Δρ_max_ = 0.34 e Å^−3^
Δρ_min_ = −0.31 e Å^−3^

### 

Data collection: *SMART* (Bruker, 2007 ▶); cell refinement: *SAINT-Plus* (Bruker, 2007 ▶); data reduction: *SAINT-Plus*; program(s) used to solve structure: *SHELXS97* (Sheldrick, 2008 ▶); program(s) used to refine structure: *SHELXL97* (Sheldrick, 2008 ▶); molecular graphics: *SHELXTL* (Sheldrick, 2008 ▶) and *PLATON* (Spek, 2009 ▶); software used to prepare material for publication: *SHELXTL*.

## Supplementary Material

Crystal structure: contains datablocks global, I. DOI: 10.1107/S1600536810036913/lh5099sup1.cif

Structure factors: contains datablocks I. DOI: 10.1107/S1600536810036913/lh5099Isup2.hkl

Additional supplementary materials:  crystallographic information; 3D view; checkCIF report

## Figures and Tables

**Table 1:** Hydrogen-bond geometry (Å, °)

*D*—H⋯*A*	*D*—H	H⋯*A*	*D*⋯*A*	*D*—H⋯*A*
O01—H1*A*⋯O1	0.92 (3)	1.93 (3)	2.854 (2)	173 (3)

**Fig. 1.**
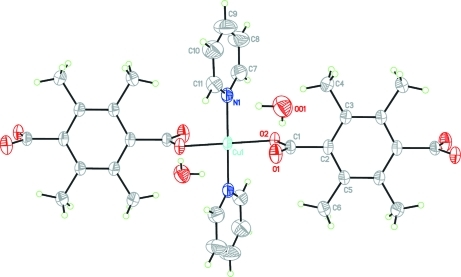
View of the coordination around the CuII ion in the title compound. Probabilty ellipsoids are drawn at the 50% level. Only the atoms of the asymmetric unit are labeled.

**Fig. 2.**
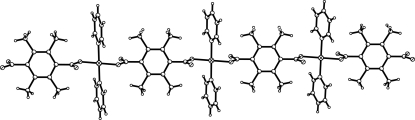
Part of the one-dimensional chain of the title compound.

**Fig. 3.**
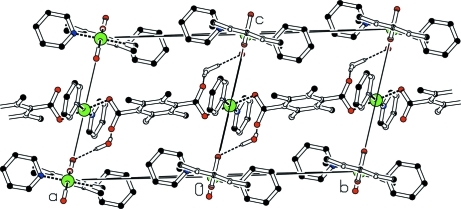
Part of the crystal structure of the title compound with hydrogen bonds shown as dashed lines. Only H atoms involved in hydrogen bonds are showm.

## supplementary crystallographic information

### Comment

The title compound (I), was designed as a ligand for preparing MOF materials and its single-crystal is presented herein. Some crystal structures containing TBDC and 1,2,4,5-benzenetetracarboxylate as ligands have already appeared in the literature (Chun *et al.*, 2005; Diniz *et al.*, 2002). The asymmetric unit (labeled in Fig. 1) contains one half copper ion, one pyridine ligand, one half solvent water molecule and half of a TBDC ligand (Fig 1.). The Cu^II^ ion lies on an inversion center and is coordinated by two oxygen atoms from two TBDC ligands and two nitrogen atoms from two pyridine ligands in a slightly distorted square-planar environment. The TBDC ligands act as bridiging to form one-dimensional chains along [110] (Fig 2.). These chains are further linked into a two-dimensional network via intermolecular O-H···O hydrogen bonds (Fig. 3).

### Experimental

A mixture of Cu(NO_3_)_2_(20 mg, 0.08 mmol),H_2_TBDC (10 mg,0.05 mmol) and two drops of pyridine was suspended in 15 ml water and heated in a teflon-lined steel bomb at 100 centigrade degree for 3 days. The block blue crystals of the title compound were obtained, washed with water and dried in the air.

### Refinement

H atoms bonded to C atoms were placed in calculated positions with C-H = 0.93-0.96Å and included in the refinement with U_iso_(H) = 1.2U_eq_(C) or 1.5U_eq_(C) for methyl H atoms. The unique H atom of the water molecule was refined indpendently with an isotropic displacement parameter. Since our goal was to prepare a porous material the solvent accessible voids of 138.00Å^3^ present in the structure might be expected.

### Crystal data

**Table:**

[Cu(C_12_H_12_O_4_)(C_5_H_5_N)_2_]·H_2_O	*F*(000) = 956
*M**_r_* = 459.98	*D*_x_ = 1.313 Mg m^−^^3^
Monoclinic, *C*2/*c*	Mo *K*α radiation, λ = 0.71073 Å
Hall symbol: -C 2yc	Cell parameters from 3713 reflections
*a* = 13.3280 (8) Å	θ = 2.4–27.5°
*b* = 17.1434 (11) Å	µ = 0.97 mm^−^^1^
*c* = 10.7390 (7) Å	*T* = 298 K
β = 108.481 (1)°	Block, blue
*V* = 2327.2 (3) Å^3^	0.15 × 0.10 × 0.08 mm
*Z* = 4	

### Data collection

**Table:**

Bruker SMART CCD diffractometer	2594 independent reflections
Radiation source: fine-focus sealed tube	2283 reflections with *I* > 2σ(*I*)
graphite	*R*_int_ = 0.017
φ and ω scans	θ_max_ = 27.5°, θ_min_ = 2.4°
Absorption correction: multi-scan (SADBAS; Sheldrick, 1996)	*h* = −17→10
*T*_min_ = 0.868, *T*_max_ = 0.926	*k* = −19→21
6747 measured reflections	*l* = −10→13

### Refinement

**Table:**

Refinement on *F*^2^	Primary atom site location: structure-invariant direct methods
Least-squares matrix: full	Secondary atom site location: difference Fourier map
*R*[*F*^2^ > 2σ(*F*^2^)] = 0.029	Hydrogen site location: inferred from neighbouring sites
*wR*(*F*^2^) = 0.086	H atoms treated by a mixture of independent and constrained refinement
*S* = 1.06	*w* = 1/[σ^2^(*F*_o_^2^) + (0.0467*P*)^2^ + 1.775*P*] where *P* = (*F*_o_^2^ + 2*F*_c_^2^)/3
2594 reflections	(Δ/σ)_max_ < 0.001
142 parameters	Δρ_max_ = 0.34 e Å^−^^3^
0 restraints	Δρ_min_ = −0.31 e Å^−^^3^

### Special details

**Table:**

Geometry. All e.s.d.'s (except the e.s.d. in the dihedral angle between two l.s. planes) are estimated using the full covariance matrix. The cell e.s.d.'s are taken into account individually in the estimation of e.s.d.'s in distances, angles and torsion angles; correlations between e.s.d.'s in cell parameters are only used when they are defined by crystal symmetry. An approximate (isotropic) treatment of cell e.s.d.'s is used for estimating e.s.d.'s involving l.s. planes.
Refinement. Refinement of *F*^2^ against ALL reflections. The weighted *R*-factor *wR* and goodness of fit *S* are based on *F*^2^, conventional *R*-factors *R* are based on *F*, with *F* set to zero for negative *F*^2^. The threshold expression of *F*^2^ > σ(*F*^2^) is used only for calculating *R*-factors(gt) *etc*. and is not relevant to the choice of reflections for refinement. *R*-factors based on *F*^2^ are statistically about twice as large as those based on *F*, and *R*- factors based on ALL data will be even larger.

### Fractional atomic coordinates and isotropic or equivalent isotropic displacement parameters (Å^2^)

**Table:**

	*x*	*y*	*z*	*U*_iso_*/*U*_eq_	
Cu1	0.0000	0.0000	0.0000	0.02763 (11)	
O2	0.07772 (9)	0.07930 (7)	−0.06903 (12)	0.0326 (3)	
N1	−0.12716 (11)	0.06855 (8)	−0.04454 (14)	0.0313 (3)	
O1	0.10913 (11)	0.10867 (8)	0.14011 (13)	0.0418 (3)	
C1	0.12054 (13)	0.12134 (9)	0.03160 (17)	0.0294 (3)	
C2	0.18792 (13)	0.18884 (9)	0.01433 (16)	0.0289 (3)	
C3	0.13967 (13)	0.25988 (10)	−0.03235 (17)	0.0312 (4)	
C5	0.29738 (13)	0.17762 (10)	0.04732 (17)	0.0315 (4)	
C11	−0.17376 (16)	0.08364 (12)	0.0463 (2)	0.0416 (4)	
H11	−0.1455	0.0619	0.1294	0.050*	
C7	−0.16747 (16)	0.10076 (13)	−0.1635 (2)	0.0451 (5)	
H7	−0.1352	0.0910	−0.2270	0.054*	
C4	0.02162 (15)	0.26957 (12)	−0.0624 (2)	0.0474 (5)	
H4A	−0.0085	0.2213	−0.0462	0.071*	
H4B	−0.0097	0.2840	−0.1528	0.071*	
H4C	0.0081	0.3096	−0.0073	0.071*	
C6	0.34512 (16)	0.09915 (12)	0.0956 (3)	0.0512 (5)	
H6A	0.2904	0.0640	0.1001	0.077*	
H6B	0.3961	0.1048	0.1813	0.077*	
H6C	0.3793	0.0788	0.0361	0.077*	
C9	−0.3036 (2)	0.16249 (18)	−0.1002 (3)	0.0691 (7)	
H9	−0.3636	0.1938	−0.1193	0.083*	
C10	−0.2619 (2)	0.13013 (15)	0.0210 (3)	0.0598 (6)	
H10	−0.2928	0.1394	0.0861	0.072*	
C8	−0.2556 (2)	0.14817 (16)	−0.1942 (2)	0.0634 (7)	
H8	−0.2823	0.1702	−0.2774	0.076*	
O01	0.0000	0.21858 (15)	0.2500	0.0601 (6)	
H1A	0.040 (2)	0.1861 (19)	0.216 (3)	0.094 (11)*	

### Atomic displacement parameters (Å^2^)

**Table:**

	*U*^11^	*U*^22^	*U*^33^	*U*^12^	*U*^13^	*U*^23^
Cu1	0.02676 (16)	0.02207 (16)	0.03625 (18)	−0.00754 (10)	0.01309 (12)	0.00071 (10)
O2	0.0348 (6)	0.0276 (6)	0.0381 (7)	−0.0120 (5)	0.0153 (5)	−0.0013 (5)
N1	0.0316 (7)	0.0265 (7)	0.0372 (8)	−0.0057 (6)	0.0128 (6)	−0.0004 (6)
O1	0.0521 (8)	0.0389 (7)	0.0396 (7)	−0.0136 (6)	0.0222 (6)	0.0006 (6)
C1	0.0286 (8)	0.0235 (8)	0.0385 (9)	−0.0048 (6)	0.0140 (7)	0.0024 (6)
C2	0.0308 (8)	0.0242 (8)	0.0332 (8)	−0.0089 (6)	0.0121 (6)	−0.0012 (6)
C3	0.0280 (8)	0.0278 (8)	0.0382 (9)	−0.0059 (6)	0.0113 (7)	0.0009 (7)
C5	0.0307 (8)	0.0245 (8)	0.0400 (9)	−0.0049 (6)	0.0120 (7)	0.0023 (7)
C11	0.0478 (11)	0.0397 (10)	0.0400 (10)	0.0014 (8)	0.0180 (8)	0.0000 (8)
C7	0.0462 (11)	0.0508 (12)	0.0397 (10)	0.0010 (9)	0.0154 (8)	0.0041 (9)
C4	0.0315 (9)	0.0413 (11)	0.0705 (14)	−0.0024 (8)	0.0178 (9)	0.0140 (10)
C6	0.0395 (10)	0.0303 (10)	0.0824 (16)	−0.0018 (8)	0.0175 (10)	0.0143 (10)
C9	0.0547 (14)	0.0729 (18)	0.0800 (18)	0.0285 (13)	0.0219 (13)	0.0116 (14)
C10	0.0622 (14)	0.0614 (15)	0.0669 (15)	0.0164 (12)	0.0364 (12)	0.0021 (12)
C8	0.0574 (14)	0.0753 (17)	0.0524 (13)	0.0182 (12)	0.0102 (11)	0.0181 (12)
O01	0.0687 (16)	0.0490 (14)	0.0683 (15)	0.000	0.0296 (13)	0.000

### Geometric parameters (Å, °)

**Table:**

Cu1—O2	1.9894 (11)	C11—H11	0.9300
Cu1—O2^i^	1.9894 (11)	C7—C8	1.380 (3)
Cu1—N1	1.9920 (15)	C7—H7	0.9300
Cu1—N1^i^	1.9921 (15)	C4—H4A	0.9600
O2—C1	1.273 (2)	C4—H4B	0.9600
N1—C11	1.337 (2)	C4—H4C	0.9600
N1—C7	1.338 (3)	C6—H6A	0.9600
O1—C1	1.241 (2)	C6—H6B	0.9600
C1—C2	1.512 (2)	C6—H6C	0.9600
C2—C3	1.394 (2)	C9—C10	1.361 (4)
C2—C5	1.401 (2)	C9—C8	1.377 (4)
C3—C5^ii^	1.401 (2)	C9—H9	0.9300
C3—C4	1.512 (2)	C10—H10	0.9300
C5—C3^ii^	1.401 (2)	C8—H8	0.9300
C5—C6	1.508 (3)	O01—H1A	0.92 (3)
C11—C10	1.374 (3)		
			
O2—Cu1—O2^i^	180.00 (6)	N1—C7—C8	121.7 (2)
O2—Cu1—N1	90.64 (5)	N1—C7—H7	119.1
O2^i^—Cu1—N1	89.36 (5)	C8—C7—H7	119.1
O2—Cu1—N1^i^	89.36 (5)	C3—C4—H4A	109.5
O2^i^—Cu1—N1^i^	90.65 (5)	C3—C4—H4B	109.5
N1—Cu1—N1^i^	180.0	H4A—C4—H4B	109.5
C1—O2—Cu1	102.52 (10)	C3—C4—H4C	109.5
C11—N1—C7	118.53 (17)	H4A—C4—H4C	109.5
C11—N1—Cu1	119.71 (13)	H4B—C4—H4C	109.5
C7—N1—Cu1	121.76 (13)	C5—C6—H6A	109.5
O1—C1—O2	122.84 (15)	C5—C6—H6B	109.5
O1—C1—C2	120.22 (15)	H6A—C6—H6B	109.5
O2—C1—C2	116.93 (14)	C5—C6—H6C	109.5
C3—C2—C5	122.37 (14)	H6A—C6—H6C	109.5
C3—C2—C1	119.25 (14)	H6B—C6—H6C	109.5
C5—C2—C1	118.37 (15)	C10—C9—C8	119.0 (2)
C2—C3—C5^ii^	118.98 (15)	C10—C9—H9	120.5
C2—C3—C4	120.19 (15)	C8—C9—H9	120.5
C5^ii^—C3—C4	120.80 (16)	C9—C10—C11	119.3 (2)
C2—C5—C3^ii^	118.64 (15)	C9—C10—H10	120.4
C2—C5—C6	120.10 (15)	C11—C10—H10	120.4
C3^ii^—C5—C6	121.25 (16)	C9—C8—C7	119.2 (2)
N1—C11—C10	122.3 (2)	C9—C8—H8	120.4
N1—C11—H11	118.9	C7—C8—H8	120.4
C10—C11—H11	118.9		
			
O2^i^—Cu1—O2—C1	93.10 (11)	C1—C2—C3—C5^ii^	179.58 (16)
N1—Cu1—O2—C1	89.16 (11)	C5—C2—C3—C4	−178.05 (18)
N1^i^—Cu1—O2—C1	−90.84 (11)	C3—C2—C5—C3^ii^	−0.2 (3)
O2—Cu1—N1—C11	−129.39 (14)	C1—C2—C5—C3^ii^	−179.59 (16)
O2^i^—Cu1—N1—C11	50.61 (14)	C3—C2—C5—C6	−179.15 (18)
O2—Cu1—N1—C7	50.15 (15)	C1—C2—C5—C6	1.5 (3)
O2^i^—Cu1—N1—C7	−129.85 (15)	C7—N1—C11—C10	0.8 (3)
Cu1—O2—C1—O1	−0.1 (2)	Cu1—N1—C11—C10	−179.66 (18)
Cu1—O2—C1—C2	179.68 (12)	C11—N1—C7—C8	−0.5 (3)
O1—C1—C2—C3	−94.9 (2)	Cu1—N1—C7—C8	179.92 (18)
O2—C1—C2—C3	85.3 (2)	C8—C9—C10—C11	−0.5 (4)
O1—C1—C2—C5	84.5 (2)	N1—C11—C10—C9	−0.3 (4)
O2—C1—C2—C5	−95.3 (2)	C10—C9—C8—C7	0.7 (4)
C5—C2—C3—C5^ii^	0.2 (3)	N1—C7—C8—C9	−0.2 (4)

Symmetry codes: (i) −*x*, −*y*, −*z*; (ii) −*x*+1/2, −*y*+1/2, −*z*.

### Hydrogen-bond geometry (Å, °)

**Table:**

*D*—H···*A*	*D*—H	H···*A*	*D*···*A*	*D*—H···*A*
O01—H1A···O1	0.92 (3)	1.93 (3)	2.854 (2)	173 (3)
